# Identification and discovery of imaging genetic patterns using fusion self-expressive network in major depressive disorder

**DOI:** 10.3389/fnins.2023.1297155

**Published:** 2023-11-21

**Authors:** Mengqian Pang, Xiaoyun Liu, Xiaoke Hao, Meiling Wang, Chunming Xie, Li Zhang, Yonggui Yuan

**Affiliations:** ^1^College of Information Science and Technology, Nanjing Forestry University, Nanjing, China; ^2^Department of Psychosomatic and Psychiatry, Zhongda Hospital, School of Medicine, Southeast University, Nanjing, China; ^3^School of Artificial Intelligence, Hebei University of Technology, Tianjin, China; ^4^School of Computer Science and Engineering, Nanjing University of Posts and Telecommunications, Nanjing, China; ^5^Department of Neurology, Zhongda Hospital, School of Medicine, Southeast University, Nanjing, China

**Keywords:** imaging genetics, major depressive disorder, multi-modality, self-expressive network, single-nucleotide polymorphisms

## Abstract

**Introduction:**

Major depressive disorder (MDD) is a prevalent mental illness, with severe symptoms that can significantly impair daily routines, social interactions, and professional pursuits. Recently, imaging genetics has received considerable attention for understanding the pathogenesis of human brain disorders. However, identifying and discovering the imaging genetic patterns between genetic variations, such as single nucleotide polymorphisms (SNPs), and brain imaging data still present an arduous challenge. Most of the existing MDD research focuses on single-modality brain imaging data and neglects the complex structure of brain imaging data.

**Methods:**

In this study, we present a novel association analysis model based on a self-expressive network to identify and discover imaging genetics patterns between SNPs and multi-modality imaging data. Specifically, we first build the multi-modality phenotype network, which comprises voxel node features and connectivity edge features from structural magnetic resonance imaging (sMRI) and resting-state functional magnetic resonance imaging (rs-fMRI), respectively. Then, we apply intra-class similarity information to construct self-expressive networks of multi-modality phenotype features via sparse representation. Subsequently, we design a fusion method guided by diagnosis information, which iteratively fuses the self-expressive networks of multi-modality phenotype features into a single new network. Finally, we propose an association analysis between MDD risk SNPs and the multi-modality phenotype network based on a fusion self-expressive network.

**Results:**

Experimental results show that our method not only enhances the association between MDD risk SNP rs1799913 and the multi-modality phenotype network but also identifies some consistent and stable regions of interest (ROIs) multi-modality biological markers to guide the interpretation of MDD pathogenesis. Moreover, 15 new potential risk SNPs highly associated with MDD are discovered, which can further help interpret the MDD genetic mechanism.

**Discussion:**

In this study, we discussed the discriminant and convergence performance of the fusion self-expressive network, parameters, and atlas selection.

## 1 Introduction

Major depressive disorder (MDD) is one of the most prevalent mental health disorders globally. It is characterized by continuous sadness, anhedonia, and changes in appetite or weight. Additionally, individuals with MDD may experience sleep disruptions, feelings of worthlessness, and thoughts of suicide. These symptoms can severely disrupt an individual's routine life and functioning (Abdoli et al., 2022). Due to its elevated incidence and recurrence rates, MDD is anticipated to become the most burdensome disease globally by 2030 (Zhang Y. et al., 2021). Nevertheless, the specific etiology of MDD remains elusive, with a non-unified pathogenic mechanism. Currently, the diagnosis of depression mainly depends on clinical symptoms and the scoring of the Hamilton Depression Rating Scale (Kennedy, 2022), which is not enough objective.

With the rapid development of imaging genetics, such as magnetic resonance imaging (MRI), functional magnetic resonance Imaging (fMRI), diffusion tensor imaging (DTI), and positron emission tomography (PET) are extensively employed for predicting and diagnosing brain and neurological disorders (Zhang et al., 2020; Li et al., 2023). Owing to its convenience and potential in assessing brain circuits, rs-fMRI become pivotal in MDD research (Liu et al., 2020b). Literature (Bondi et al., 2022; Dai et al., 2022) suggested that using machine learning with rs-fMRI could effectively diagnose MDD and pinpoint significant features. Moreover, combining functional connectivity (FC) with network attributes helped in distinguishing between MDDs and healthy controls (HCs). Despite their promise to identify dynamic FC changes, these methods often rely solely on a single imaging modality, limiting their capacity to find consistent biomarkers across multiple imaging data.

In recent years, imaging genetics provided vast opportunities for examining the influence of genetic variations on brain structure and function. Building on this concept, the primary objective in this field was to measure the association between genetic variations, such as SNPs, and neuroimaging biomarkers derived from various imaging modes. These insights deepened our understanding of the intricate pathogenesis of diseases (Hao et al., 2016). Given that the Alzheimer's Disease Neuroimaging Initiative (ADNI) database encompassed multi-modality imaging and genotype data, a significant portion of brain imaging genetic research was focused on Alzheimer's disease (Windon et al., 2022). However, few studies employed machine learning techniques to explore neurogenetic correlations specific to MDD. Zhang et al. (2023) proposed a methodology that integrates multi-stage diagnosis status to unearth associations between genetic risk variants and the multi-modality phenotype network in MDD. This approach successfully identified the risk SNP THP1 rs1799913 as being highly associated with MDD. Nevertheless, it overlooked the intricate spatial structure of multi-modality data, suggesting a potential for further refinement.

To address this, we introduced a self-expressive network (Elhamifar and Vidal, 2013; Ji et al., 2017). To theorize, each data point is distributed within a union of subspaces, and these subspaces can effectively represent the linear or affine combinations of samples in the dataset that belong to the same subspace. These subspaces were determined by clustering samples, where similar samples clustered in the same subspace, and dissimilar samples were distributed in different subspaces. First, we proposed a fusion self-expressive network that leveraged both participant diagnosis information and local network structures for correlation investigations. Then, we developed an imaging-genetics analysis framework, calculating relationships among subjects within the same class and constructing a self-expressive network of imaging-genetics data through sparse reconstruction. Finally, by incorporating participant diagnosis information, different self-expressive networks were iteratively merged using a fusion approach, forming a network capable of representing the underlying data structure. Our method was validated using the MDD genetic risk SNP TPH1 rs1799913. Experimental results indicated that our method not only improved the performance of correlation coefficient (CC) metrics but also identified a concise set of common ROIs shared between two brain network features closely associated with the MDD genetic risk SNP TPH1 rs1799913. Furthermore, we successfully identified 15 new potential risk SNPs associated with MDD, which also identified several MDD-related ROIs.

The contributions of this paper are as follows:

1. To effectively extract similar structures within the data, we introduced a self-expressive network for self-reconstruction of the original data.
2. We proposed a fusion self-expressive network that incorporated participant diagnosis information. This approach allowed for iterative fusion, resulting in a unified network that captured the comprehensive structure of the underlying data.
3. An association model based on the fusion self-expressive network was developed to mine the risk SNPs and multi-modality brain phenotype network. Using the risk SNP rs1799913 to validate our method, experimental results demonstrated its superiority over existing algorithms.
4. We also identified 15 new potential risk SNPs associated with MDD, providing a foundation for researchers to further investigate the mechanisms underlying MDD pathogenesis.

The subsequent sections of this paper are structured as follows: Section 2 presents the demographic statistics of participants and the pre-processing of multi-modality data. In Section 3, the proposed methodology for the identification of risk-associated SNPs and the construction of the multi-modal phenotype network is introduced. The introduction and analysis of experimental results are found in Section 4. Lastly, discussions and conclusions are respectively presented in Sections 5 and 6.

## 2 Data source and preprocessing

### 2.1 Participants

This study utilized datasets obtained from two hospitals, namely the Affiliated Zhongda Hospital of Southeast University and the Second Affiliated Hospital of Xinxiang Medical University. Patients were recruited from the inpatient and outpatient departments of psychiatry in these hospitals, while HCs were recruited through media advertising and community posting. The research procedures followed the guidelines outlined in the Declaration of Helsinki. All patients provided informed consent and met the following inclusion criteria: (1) met the diagnostic criteria outlined in the Diagnostic and Statistical Manual of Mental Disorders (Fourth Edition); (2) experienced their first depressive episode and were above 18 years old at onset; (3) obtained Hamilton Depression Scale-24 (HAMD-24) scores ≥20; (4) had no history of major psychiatric illness other than depression; (5) had no primary neurodegenerative disorders such as dementia or stroke; (6) had no history of substance abuse or dependence (including drugs, caffeine, nicotine, alcohol, or others), head trauma, or loss of consciousness; (7) had no cardiac or pulmonary diseases that could influence the MRI scan. The HC subjects met criteria (4) to (7) and were required to have a HAMD-24 score ≤ 8.

After excluding low-quality images affected by head motion or ghost intensity artifacts, this study included a total of 26 HCs and 45 patients with MDD from the Affiliated ZhongDa Hospital of Southeast University, as well as 38 HCs and 62 MDD patients from the Second Affiliated Hospital of XinXiang Medical University. The severity of depression was assessed using the Hamilton Depression Scale-24 (HAMD-24) scores. Among the 107 MDD patients included in the study (with HAMD-24 scores ≥20), they were further categorized into two subgroups based on their HAMD-24 scores: moderate depression (MD) for scores ranging from 20 to 34, and severe depression (SD) for scores ≥35 (Tolentino and Schmidt, 2018). The demographic characteristics of the participants are presented in Table 1.

**Table 1:** Demographic statics of subjects.

**Hospital**	**ZhongDa**			**XinXiang**		
**Subject**	**HC**	**MD**	**SD**	**HC**	**MD**	**SD**
Number	26	34	11	38	44	18
Gender (M/F)	10/16	15/19	4/7	21/17	26/18	7/11
Age (mean ± std)	36.69 ± 13.88	44.94 ± 14.51	44.09 ± 15.22	44.76 ± 12.25	42.16 ± 13.26	47.44 ± 14.11
Education (mean ± std)	13.46 ± 3.96	11.09 ± 4.14	10.09 ± 5.99	10.74 ± 4.67	9.89 ± 4.25	9.44 ± 4.19
HAMD-24 (mean ± std)	1.27 ± 2.16	27.65 ± 4.68	38.91 ± 2.26	1.13 ± 1.85	29.91 ± 3.66	39.22 ± 3.95

HC, healthy control; MD, moderate depression; SD, severe depression; M/F, male/female; HAMD-24, Hamilton Depression Scale-24.

### 2.2 Magnetic resonance imaging data acquisition and preprocessing

All participants underwent baseline magnetic resonance imaging (MRI) scans using a 3.0 T Siemens scanner (Siemens, Erlangen, Germany) with a 12-channel head coil. Pads were used to immobilize the heads of all subjects and minimize head movements. High-resolution 3D T1-weighted scans were acquired using a magnetization-prepared fast gradient echo (MPRAGE) sequence with the following parameters: repetition time (TR) = 1,900 ms, echo time (TE) = 2.48 ms, flip angle (FA) = 9°, acquisition matrix = 256 *times* 256, field of view (FOV) = 250 × 250 m^2^, slice thickness = 1.0 mm, no gap between slices, scan time = 4 min 18 s, and a total of 176 volumes.

For the rs-fMRI scans, the following parameters were used: TR = 2,000 ms, TE = 25 ms, FA = 90°, acquisition matrix = 64 × 64, FOV = 240 × 240 *m*^2^, slice thickness = 3.0 mm, no gap between slices, axial slice orientation with 36 slices, a total of 240 volumes, in-plane resolution parallel to the anterior-posterior conjunction = 3.75 × 3.75 m^2^, and an acquisition time of 8 min. During the scans, subjects were instructed to lie on their backs with their hands naturally resting on their sides. Head movement was minimized by using pads, and ear plugs were provided to reduce scanner noise. Subjects were asked to relax their bodies, keep their eyes open, stay awake, and avoid focusing on any specific thoughts to prevent falling asleep. Image quality was checked immediately after scanning, and repeat scans were performed if necessary.

For quality control, two experienced radiologists examined all image data. The rs-fMRI images were preprocessed using the Resting State Functional Data Processing Assistant (DPARSF 2.3 Advanced) MRI toolkit, which integrates the Resting State Functional MRI Toolkit (REST) and the Statistical Parametric Mapping Package (SPM) programs (Yan and Zang, 2010). The first 10 time points were excluded to ensure stable longitudinal magnetization and accommodate scanner noise. The remaining 230 images underwent sequential processing steps: (1) correction for time differences and head motion using the 36th slice as the reference slice for slice time correction (participants with maximum head motion displacement >1.5 mm in any direction or angular motion >1.5° were excluded); (2) co-alignment of the T1-weighted image with the functional image and subsequent reorientation; (3) spatial normalization, where the T1-weighted anatomical images were segmented into white matter, gray matter, and cerebrospinal fluid, and then normalized to the Montreal Neurological Institute (MNI) space using a uniform segmentation algorithm. The transform parameters from the segmentation were applied to the functional images, which were resampled with 3 mm isotropic voxels; (4) spatial smoothing with a 4 mm full-width at half-peak (FWHM) isotropic Gaussian kernel; removal of linear trends within each voxel time series; regression of interference signals (white matter, cerebrospinal fluid signals, and head motion parameters calculated using rigid body six corrections); removal of spiked regression volumes; and application of a temporal bandpass filter (0.01–0.08 Hz) to reduce low-frequency drift and filter out high-frequency noise.

### 2.3 SNP genotype data sequencing and processing

DNA genotyping was conducted by Tianhao Biotechnology (Shanghai, China), following the standard protocol for DNA extraction from blood samples. Illumina Next sequencing and array technology (Illumina Inc., San Diego, CA, USA) were employed to determine the genotypes of single nucleotide polymorphisms (SNPs) in the genes of interest. Subsequently, we utilized the PLINK (v1.9) software to perform the Hardy-Weinberg equilibrium (HWE) test, assess linkage disequilibrium statistics, and calculate allele and genotype frequencies (Purcell et al., 2007). After excluding missing or erroneous values, a total of 5,897 SNPs were retained for further analysis in this study.

Genetic risk variants can help researchers understand the biological mechanisms of related diseases and provide valid hypotheses for drug design. In this study, we focused on thoroughly exploring the relationship between a given risk SNP and quantitative traits at the level of brain structure and function. Some studies have implicated a large number of genes associated with depression, including HTR2A, CACNA1C, BDNF, CRHR1, GSK3β, TPH1, among others, as detailed in a systematic review (Flint and Kendler, 2014). However, unlike the well-known risk SNP APOE rs429358 in Alzheimer's disease, there was no consistent genetic hypothesis for the pathogenesis of MDD. Tryptophan hydroxylase (TPH) was the rate-limiting enzyme in the biosynthesis of serotonin (5-HT). Haplotype analysis suggested a connection between TPH-1 and MDD. In this study (Zhang et al., 2023), TPH1 rs1799913 was found to have a strong correlation with MDD. Gizatullin et al. (2006) discovered six SNPs that were in linkage disequilibrium in both the patient and control groups. However, only one SNP (rs1799913) was significantly associated with MDD in their single-marker association analysis. This SNP was also referenced as SNP A779C in other research. It was linked to the cerebrospinal fluid (CSF) 5-hydroxyindoleacetic acid (5-HIAA) concentration, and multiple studies had indicated its association with suicidal behavior (Jarienė et al., 2018; Nielsen et al., 2020).

Therefore, we first used TPH1 rs1799913 to validate our proposed model. The values of TPH1 rs1799913 were encoded additively as 0, 1, 2, where the alleles were divided into major and minor alleles based on genotype frequencies, with the major allele encoded as “0,” the minor allele as “2” and the heterozygotes as “1” (Purcell et al., 2007). After validation, the remaining 5,896 SNPs were encoded using the same method and subsequently analyzed to determine their correlations with MDD.

## 3 Method

**Figure 1:**
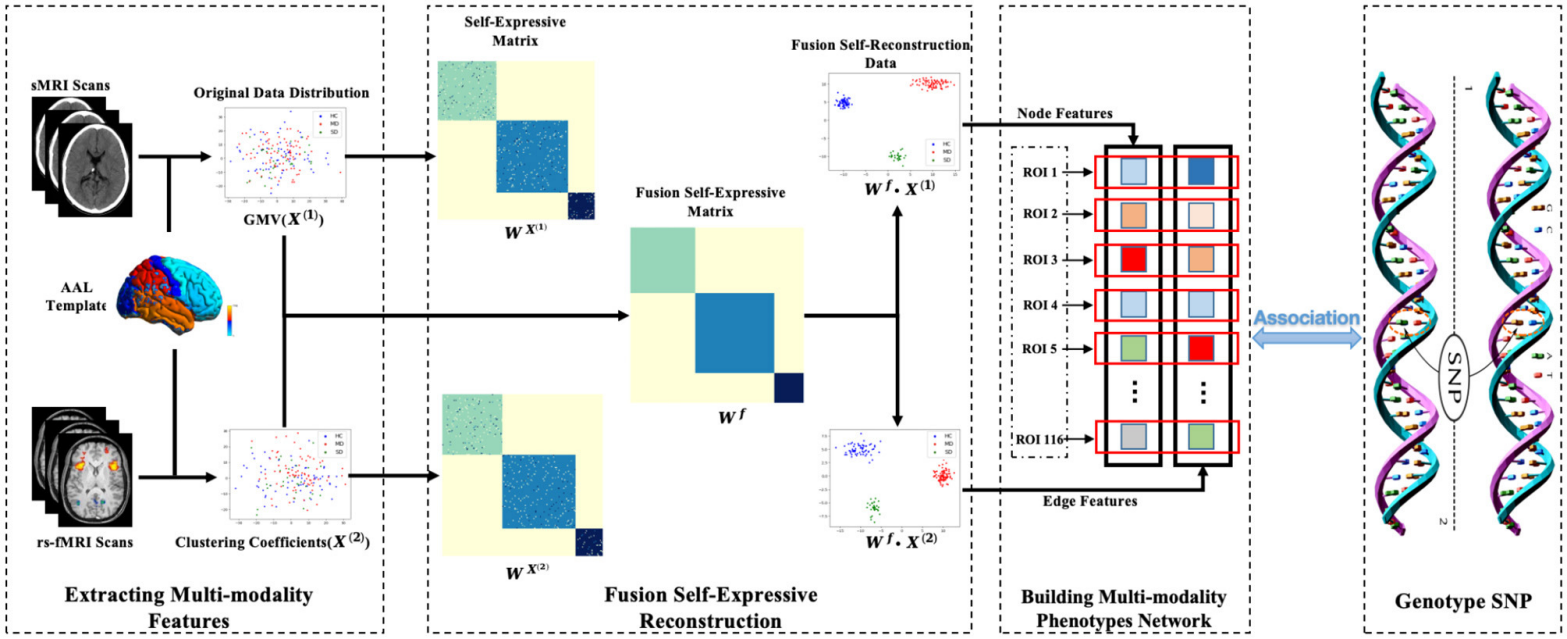
The overview of FSN-MM model.

To simultaneously focus on brain structural imaging and functional connectivity information between different brain regions while considering the complex internal data structure, we proposed the FSN-MM model by diagnosis information to mine the relationship between MDD risk SNPs and the multi-modality phenotype network. The overview of our proposed model is depicted in Figure 1. We utilized the Automated Anatomical Labeling (AAL) atlas to extract voxel node features from sMRI data and network connectivity edge features from rs-fMRI data, respectively. Subsequently, we employed intra-class similarity information to construct novel self-expressive networks for the multi-modality phenotype by sparse representation. These self-expressive networks were then iteratively merged into a single network guided by diagnosis information. Leveraging this fusion self-expressive network model, we obtained a new restructured multi-modality phenotype network and applied a multi-modality association model to investigate the relationship between MDD risk SNPs and the multi-modality phenotype network.

### 3.1 Extracting the features of multi-modality phenotype network

The node and edge features of each subject were integrated to form a multi-modality brain phenotype network. The node features were extracted from sMRI data, while the edge features were extracted from rs-fMRI data.

After preprocessing the sMRI data, voxel-based morphometry (VBM) analysis was conducted on each subject. This analysis utilized normalized gray matter density masks with voxel dimensions of 2 × 2 × 2 mm^3^ in the MNI space. The resulting VBM outputs were registered to the respective patient scans for each participant, and the mean gray matter volume (GMV) measurements were extracted for 116 ROIs based on the AAL template. Each ROI was considered as an individual node within the multi-modality brain network, enabling the derivation of a set of node features for each subject.

A functional connectivity network was established for each individual using rs-fMRI data, where nodes represented predetermined ROIs and edges signified the functional connections between these ROIs. The average time series of each ROI, normalized to have zero mean and unit variance, were extracted based on the AAL template. The functional connectivity networks were derived using the Pearson correlation coefficient, which captured the correlation between BOLD signals of paired ROIs (Li et al., 2017). Graph theory-based methods played a crucial role in the analysis of human brain disorders due to the abnormal topological properties of brain networks in psychiatric disorders. One of the most commonly used methods was clustering coefficients, which reflected the local clustering properties of the brain network (Liu J. et al., 2020; Li et al., 2022). The clustering coefficient (CC) is a threshold-free metric used to measure the degree of node aggregation in network graph theory and is one of the most commonly used methods. The CC for each ROI is determined by the functional connectivity between that ROI and other ROIs.

### 3.2 Self-expressive network

The concept of self-expressiveness postulates that each data point is distributed across multiple subspaces, and each subspace can be accurately represented by a linear or affine combination of samples from the same subspace. Within the multi-modality phenotype network, a complex structure is formed, encompassing several subspaces. Our methodology leverages this inherent self-expressiveness property, which captures the similarity structure of the data, to reconstruct the original input before performing correlation analysis. By introducing a self-expressive network for self-reconstruction, we can comprehensively describe the similarities present within the data. To construct such a self-expressive network, we utilize the self-expressiveness property to create a subject-subject similarity relationship matrix. This matrix serves as an equivalent representation of the self-expressive network, where the weighted edges signify pairwise similarity relationships.

In a self-expressive network *SN* = {*V, E, W*}, *V* represents a collection of nodes where each node corresponds to a subject. *E* represents a set of edges, and *W* represents a weight matrix for the edges. Specifically, given a set of data points {_*x*_*i*_}*i* = 1, ⋯, *N*_ derived from multiple linear subspaces {_*R*_*i*_}*i* = 1, ⋯, *K*_, it is feasible to express a point in a specific subspace as a linear combination of other points in the same subspace. For the data matrix *X*, the property of self-expressiveness can be represented as *X* = *W*^*x*^*X*, with *X* = *W*^*x*^*X* being the self-expressive coefficient matrix. As shown in Ji et al. (2017), under the assumption of independence of the subspaces, the minimization of certain norms of *W*^*x*^ ensures a block-diagonal structure of *W*^*x*^ (up to some permutations). That is, $W_{ij}^{x}\neq 0$ only if points *x*_*i*_ and *x*_*j*_ reside in the same subspace. In mathematical terms, this can be formulated as an optimization problem:


(1) $$\begin{array}{l} \begin{array}{cc} \underset{W^{x}}{min}||W^{x}|{|}_{p} & s.t.X=W^{x}X,\left(diag\left(W^{x}\right)=0\right) \end{array} \end{array}$$

The weight matrix in the sparse representation reflects the inherent geometric properties of the data and *W*^*x*^ should have a block-diagonal structure under a certain permutation, where each block corresponds to the data samples from the same subspace.

The aforementioned problem can also be approached as identifying clustering structures or block structures within the weight matrix *W*^*x*^ that correspond to the subspaces of the data. This approach is equivalent to leveraging the self-expressive property, which captures the similarity structure of the data, for reconstructing the original data. In this study, we utilize diagnosis information (i.e., HC, MD, SD) to perform the data reconstruction. Specifically, given *c* classes of samples $\tilde{X}=\left[x_{1}^{\left(1\right)},\cdots \;,x_{N_{1}}^{\left(1\right)},\cdots \;,x_{1}^{\left(c\right)},\cdots \;,x_{N_{c}}^{\left(c\right)}\right]\in R^{p\times N}$, we reconstruct *x*_*i*_ using samples with the same label. Here, *p* represents the number of ROIs, $x_{i}^{\left(c\right)}$ represents the *i*-th sample in the *c*-th class, and it can be expressed as a combination of samples with the same diagnostic label, as shown in Equation 2:


(2) $$\begin{array}{l} \underset{{\left(W_{i}^{x}\right)}^{\left(c\right)}\geq 0}{min}\frac{1}{2}||x_{i}^{\left(c\right)}-{\left(\tilde{X}\right)}^{\left(c\right)}{\left(W_{i}^{x}\right)}^{\left(c\right)}|{|}_{2}^{2}+\lambda ||{\left(W_{i}^{x}\right)}^{\left(c\right)}|{|}_{1} \end{array}$$

where λ denotes a regularization parameter. ${\left(W_{i}^{x}\right)}^{\left(c\right)}$ is a vector in which the element corresponding to $x_{i}^{\left(c\right)}$ is zero, and we discard negative solutions. Specifically, ${\left(W_{i}^{x}\right)}^{\left(c\right)}={\left[0,\cdots \;,0,W_{i,1},\cdots \;,W_{i,i-1},0,W_{i,i+1},\;cdots,W_{i,N_{j}},0,\cdots \;,0\right]}^{T}$. Subsequently, we utilize the SLEP toolbox (Liu et al., 2009) to optimize Equation 2. Once we obtain ${\left(W_{i}^{x}\right)}^{\left(c\right)}$, the optimal solution of Equation 2, we can express the sparse reconstructive weight matrix *W*^*x*^ as follows:


(3) $$\begin{array}{l} W^{x}=\left[{\left({\tilde{W}}_{1}^{x}\right)}^{\left(c\right)},...,{\left({\tilde{W}}_{N}^{x}\right)}^{\left(c\right)}\right]+{\left[{\left({\tilde{W}}_{1}^{x}\right)}^{\left(c\right)},...,{\left({\tilde{W}}_{N}^{x}\right)}^{\left(c\right)}\right]}^{T} \end{array}$$

**Figure 2:**
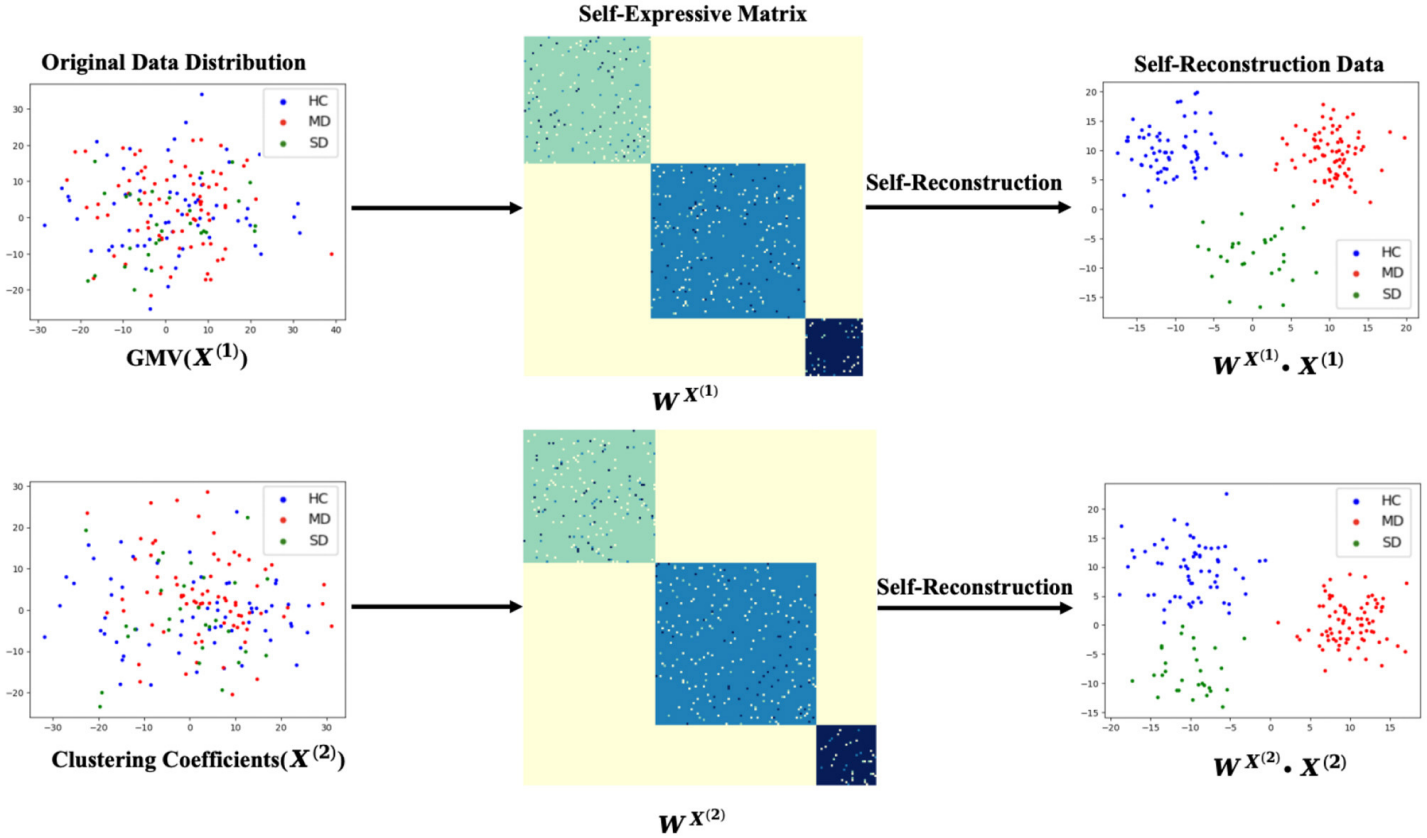
The process of data self-reconstruction by self-expressive method.

The reconstructive weight matrix *W*^*x*^ is an *N*×*N* symmetric matrix, where each element represents the contribution of each *x*_*j*_ to the reconstruction of *x*_*i*_. The diagonal elements of the constructed matrix *W*^*x*^ are set to zero. As shown in Figure 2, the self-reconstruction data demonstrates some clustering effects compared to the original data.

### 3.3 Fusion self-expressive network

The utilization of multi-modality brain imaging phenotypes is widely accepted as it provides more comprehensive information compared to single-modality biomarkers. With the availability of multi-modality data, fusion methods based on sample similarity have been extensively employed in various tasks such as classification, clustering, and prediction (Wang et al., 2014). Drawing inspiration from these previous studies and enhancing the clustering accuracy by bringing together data diagnosed within the same class in the multi-modality phenotype network, we introduce a fusion self-expressive network. This network effectively leverages the local structure of the network and diagnosis information between networks to facilitate efficient data integration.

The fusion self-expressive network *FSN* = {*V*^*f*^, *E*^*f*^, *W*^*f*^} is defined as the network that incorporates fusion self-expressiveness, which includes high-weight edges, from one or more of these networks. In this context, the self-expressiveness property can be represented as *X*^*m*^ = *W*^*f*^*X*^*m*^, where *X*^*m*^ denotes the multi-modality brain imaging phenotypes and *W*^*f*^ represents the fusion self-expressive coefficient matrix. Specifically, given *M* self-expressive networks *SN*^*m*^ = {*V*^*m*^, *E*^*m*^, *W*^*m*^} derived from *M* multi-modality brain imaging phenotypes $X^{m}={\left[x_{1}^{m},\cdots \;,x_{n}^{m},\cdots \;,x_{N}^{m}\right]}^{T}\in R^{N\times r}\left(m=1,2,\cdots \;,M\right)$, *r* represents the number of ROIs. We aim to obtain the fusion self-expressive coefficient matrix *W*^*f*^ from the self-expressive network matrices *W*^*m*^. To accomplish this objective, we compute the local affinity of the self-expressive network matrix based on diagnosis information. This operation involves setting the similarity (calculated as pairwise similarity values) between points of different classes to zero. Essentially, we assume that pairwise similarities with high weight values are within the same class more reliable than similarities between different classes. Therefore, we assign similarities to points of different classes through graph diffusion on the network. The calculation of the local affinity (*A*_*L*_) can be expressed as follows:


(4) $$A_{L}^{m}(i,j)=\{\begin{array}{cc} \frac{W^{m}(i,j)}{\sum_{k\in C_{i}}W^{m}(i,k)} & j\in C_{i}\\ 0 & otherwise \end{array}$$

where *C*_*i*_ represents the set of points in the same class as *x*_*i*_ in *SN*^*m*^. Subsequently, for *M* self-expressive networks, the updated self-expressive matrix for the multi-modality data can be defined as:


(5) $$\begin{array}{l} W_{t+1}^{m}\left(i,j\right)=\underset{k\in C_{i}}{\sum }\underset{l\in C_{i}}{\sum }A_{L}^{m}\left(i,k\right)A_{L}^{m}\left(j,l\right)\frac{\underset{q\neq m}{\sum }W_{t}^{q}\left(k,l\right)}{M-1} \end{array}$$

where $W_{t+1}^{m}$ represents the fusion self-expressive network matrix after *t* iterations. The matrix *W* contains the complete information about the similarity of each subject to all others in the dataset. In contrast, the matrix *A*_*L*_ focuses solely on encoding the similarity between a given patient and others within the same class. Our algorithm always starts with *W* as the initial state and utilizes *A*_*L*_ as the kernel matrix in the fusion iteration process to capture the local structure of networks (Wang et al., 2021). The self-expressive information between subjects of the same class is only propagated through the common class. Furthermore, it is worth mentioning that if *x*_*i*_ and *x*_*j*_ are dissimilar in one modality, the self-expressive information can be expressed in other modalities. Thus, the self-expressive information among subjects within the same class can be obtained through fusion. Ultimately, after *t* iterations, the overall fusion self-expressive network matrix can be formulated as:


(6) $$W^{f}=\{\begin{array}{cc} \frac{1}{M}\sum_{m=1}^{M}W_{t}^{m}(i,j) & i\neq j\\ 0 & i=j \end{array}$$

Equation 6 demonstrates that the fusion self-expressive network effectively leverages the local structure within networks and captures both common and complementary information across multiple modalities. The self-expressiveness property is expressed as *X*^*m*^ = *W*^*f*^*X*^*m*^, where *W*^*f*^ represents the fusion self-expressive coefficient matrix.

**Figure 3:**
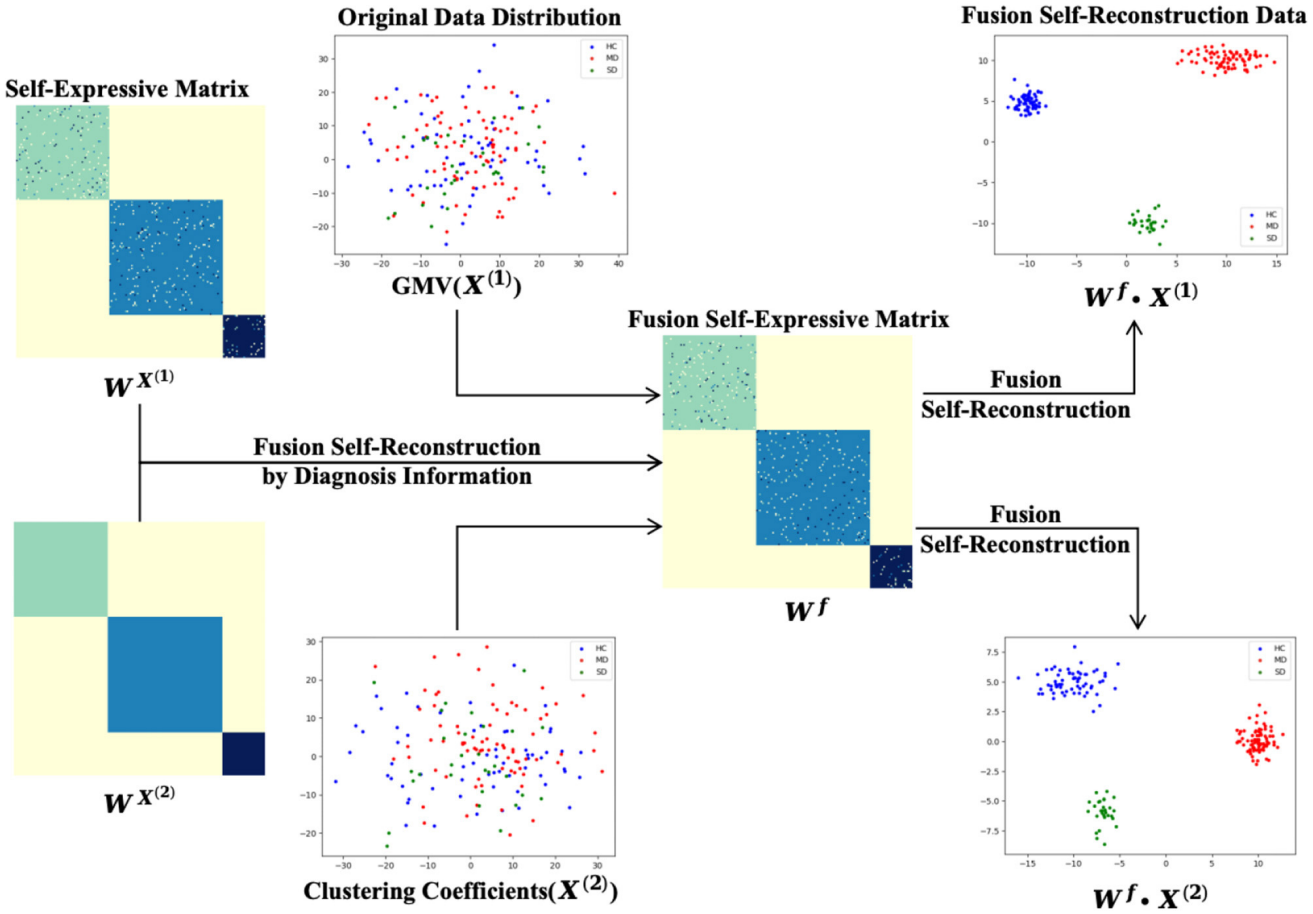
The process of data fusion self-reconstruction and fusion of self-expressive matrices.

Figure 3 illustrates the process of self-expressive network fusion and the data fusion self-reconstruction process. Specifically, the model iteratively integrates self-expressive networks from the multi-modality phenotypes network into a single network, guided by diagnosis information. During the fusion process, weak similarities (low-weight edges) are eliminated, while strong similarities (high-weight edges) emerge within one or more networks and are incorporated into other networks. Additionally, low-weight edges supported by all networks are preserved, depending on their connectivity strength across classes spanning multiple networks. In the fusion self-expressive matrix, the intra-class similarity (high-weight edges) is enhanced, while the inter-class similarity (low-weight edges) is significantly reduced. By comparing with Figure 2, it is evident that the fusion self-reconstruction data exhibits better clustering performance than the self-reconstruction data.

### 3.4 Introduction fusion self-expressive network into association model

Once the multi-modality phenotype network is constructed for each subject, pathological changes can be identified as abnormal alterations within the phenotype networks. These changes are closely associated with relevant ROIs and significant connectivity edges, as the features of the network nodes and edges are derived from GMV data obtained from sMRI and clustering coefficients from rs-fMRI. Here, let's consider a scenario with *N* subjects, and let each subject represent itself with a multi-modality phenotype network. The input consists of *M* modalities of phenotypes, denoted as $X^{m}={\left[X_{1}^{m},\cdots \;,X_{n}^{m},\cdots \;,X_{N}^{m}\right]}^{T}\in R^{N\times d}$, where *d* represents the dimensionality of node and edge features. The corresponding output is represented by $y={\left[y_{1},\cdots \;,y_{n},\cdots \;,y_{N}\right]}^{T}\in R^{N}$, where *y* represents the response value of SNP. In this context, let *w*^*m*^∈*R*^*d*^ denote the linear discriminant function corresponding to the *m*-th modality. The multi-modality network phenotype association model can be formulated as:


(7) $$\begin{array}{l} \underset{W}{min}\frac{1}{2}\overset{M}{\underset{m=1}{\sum }}||y-X^{m}w^{m}|{|}_{2}^{2}+\lambda ||W|{|}_{2,1} \end{array}$$

where *W* = [*w*^1^, *w*^2^, ⋯, *w*^*M*^]∈*R*^*d*×*M*^ is the weight matrix, and each row *w*_*j*_ represents the vector of coefficients assigned to the*j*-th feature across multi-modality. It is important to note that Equation 7 introduces the *L*_2, 1_-norm regularization term, $||W|{|}_{2,1}=\overset{d}{\underset{j=1}{\sum }}||w_{j}|{|}_{2}$, which serves as a “group-sparsity” regularizer. This regularization term penalizes all coefficients in the same row of the matrix *W*, encouraging joint feature selection. In other words, our proposed model aims to select only a small number of features across multi-modality. The regularization parameter λ controls the balance between the two terms in Equation 7. A larger value of λ leads to the selection of fewer features.

Then, we incorporate the fusion self-expressive network into the multi-modality association model by incorporating Equation 6 into Equation 7. The objective function of our proposed association model, referred to as FSN-MM, can be formulated as follows:


(8) $$\begin{array}{l} \underset{W}{min}\frac{1}{2}\overset{M}{\underset{m=1}{\sum }}||y-W^{f}X^{m}w^{m}|{|}_{2}^{2}+\lambda ||W|{|}_{2,1} \end{array}$$

where the parameter λ controls the regularization term, and its value can be determined through inner cross-validation on the training data. In the objective function (Equation 8), we replace the original data with the fusion self-reconstructed data. With this formulation, the FSN-MM model can jointly select a sparse subset of common features from the multi-modality data while fully leveraging the prior diagnosis information among subjects.

## 4 Experimental results and analysis

### 4.1 Experimental settings

In our experiments, we utilized the CC as an evaluation metric to measure the association analysis between the predicted and actual response values and employed the five-fold cross-validation strategy to validate the effectiveness of our proposed method. To determine the parameter λ in Equation 8, we tuned its values from 10^−5^, 3 × 10^−5^, 10^−4^, 3 × 10^−4^, ⋯, 3 and selected the optimal value through nested five-fold cross-validation on the training dataset.

In Table 2, we compared the performance of the single-modality (SM) method, concatenate-modality (CM) method, and multi-modality (MM) method with/without fusion self-expressive network. The SM, CM, and MM methods represented conventional approaches without incorporating the fusion self-expressive network. We presented the FSN-SM and FSN-CM methods, which were the improved versions of the SM and CM methods incorporating the fusion self-expressive network, respectively. The FSN-MM method simultaneously considered the multi-modality images and fusion self-expressive network. Additionally, we presented the SN-SM, SN-CM, and SN-MM methods, which were the improved versions of the SM, CM, and MM methods incorporating the self-expressive network, respectively. Descriptions of the various comparisons were presented in Table 2.

**Table 2:** The detailed description of various comparisons.

**Comparison**	**Modality**	**Diagnosis**	**Description**
SM	Node	No	Employing the least absolute shrinkage and selection operator (LASSO) technique, a sparse, yet significant, subset from either node or edge features is identified
	Edge	No	
SN-SM	Node	Yes	
	Edge	Yes	
FSN-SM	Node	Yes	
	Edge	Yes	
CM	–	No	By concatenating node and edge features, a subsequent application of the LASSO allows for the detection of a sparse yet significant subset within the merged features
SN-CM	–	Yes	
FSN-CM	–	Yes	
MM	Node	No	Identifying a sparse subset of shared ROIs derived from node and edge features
	Edge	No	
SN-MM	Node	Yes	
	Edge	Yes	
FSN-MM	Node	Yes	
	Edge	Yes	

### 4.2 Identification of imaging-genetic patterns in MDD using FSN-MM

#### 4.2.1 Association between risk SNP and multi-modality network phenotype

We validated the model using the MDD risk SNP rs1799913. In our comparison, we evaluated the performance of our proposed FSN-MM method and compared it with conventional methods (including SM, CM, and MM) as well as improved methods that incorporate the fusion self-expressive network (including FSN-SM and FSN-CM). To ensure unbiased results, we performed five independent and non-repetitive five-fold cross-validations. We calculated the average CC results on the training and testing data separately for the node and edge modalities. Table 3 presented the average results.

**Table 3:** Comparison of regression performance on risk SNP TPH1 rs1799913 by different methods.

**Method**		**CC (mean** ±**SD)**	
		**Train**	**Test**
SM	Node	0.0465 ± 0.0440	0.0121 ± 0.0045
	Edge	0.0983 ± 0.0564	0.0488 ± 0.0027
SN-SM	Node	0.2125 ± 0.0538	0.1558 ± 0.0571
	Edge	0.2013 ± 0.0148	0.1347 ± 0.0777
FSN-SM	Node	0.3055 ± 0.0370	0.1934 ± 0.0509
	Edge	0.3156 ± 0.0982	0.2031 ± 0.0283
CM	–	0.1258 ± 0.0415	0.0875 ± 0.0842
SN-CM	–	0.2964 ± 0.0375	0.1603 ± 0.0065
FSN-CM	–	0.3702 ± 0.0861	0.2354 ± 0.0175
MM	Node	0.5416 ± 0.0412	0.1654 ± 0.0218
	Edge	0.4645 ± 0.0561	0.1714 ± 0.0544
SN-MM	Node	0.3885 ± 0.0079	0.2528 ± 0.0860
	Edge	0.3678 ± 0.0093	0.2306 ± 0.0449
FSN-MM	Node	0.5401 ± 0.0661	0.3845 ± 0.0040
	Edge	0.5634 ± 0.0263	0.4037 ± 0.0095

The bold values are the values of the method we proposed.

As shown in Table 3, FSN-SM achieves CC values of 0.1934 and 0.2031 on the node and edge features, respectively, outperforming the conventional SM method. FSN-CM achieves a CC value of 0.2354, which is an improvement over the CM method. FSN-MM demonstrates the best performance with CC values of 0.3845 and 0.4037 on the two different features. These results highlight several important findings:

1) MM-type methods, such as FSN-MM, outperform SM-type methods by jointly selecting node and edge features, leading to enhanced performance in association analysis.
2) The introduction of the fusion self-expressive network, incorporating diagnosis information, consistently improves the CC performance compared to conventional methods across different feature types.
3) Functional connectivity edge features between different brain regions, compared to voxel-based morphometry node features, provide more insights for understanding the mechanisms of MDD.

Moreover, CM-type and MM-type methods both utilize node and edge features, but they employ different strategies to combine these features, resulting in distinct performance. CM-type methods directly concatenate node and edge features, which may lead to the loss of relationship information between the modalities and introduce more noise in the expanded feature space. On the other hand, MM-type methods utilize the multi-task strategy with *L*_2, 1_-norm constraint to jointly select node and edge features, improving the robustness of ROI detection.

In summary, the FSN-MM method demonstrates the best performance in terms of CC measure. This indicates that considering multi-modality imaging data and incorporating the fusion self-expressive network with diagnosis information can effectively enhance the performance of association analysis between imaging phenotypes and genotypes.

#### 4.2.2 Comparison with state-of-the-art approach

Based on existing MDD research, this study utilizes multi-modality data and compares the FSN-MM method with the state-of-the-art imaging genetics algorithm for MDD, known as MSD-MM (Zhang et al., 2023), as shown in Table 4. To ensure the validity and reliability of our proposed approach, the datasets, risk gene SNP (TPH1 rs1799913), and processing framework (including templates) used in this study are consistent with those in the research (Zhang et al., 2023).

**Table 4:** Performance comparison with state-of-the-art approach on SNP rs1799913.

**Method**	**CC (mean** ±**SD)**	
	**Node**	**Edge**
MSD-MM	0.2432 ± 0.0799	0.2697 ± 0.0910
FSN-MM	**0.3845** **±0.0040**	**0.4037** **±0.0095**

The bold values are the values of the method we proposed.

The CC values for FSN-MM on node and edge features are 0.3845 and 0.4037, respectively. In contrast, the CC values for MSD-MM on these features are 0.2432 and 0.2697, respectively. The FSN-MM method outperforms the MSD-MM algorithm in imaging genetics association. This study provides a practical solution for constructing and utilizing the fusion self-expressive network to build an imaging genetics analysis framework.

#### 4.2.3 Identification of the consistent ROIs from multi-modality imaging data

**Figure 4:**
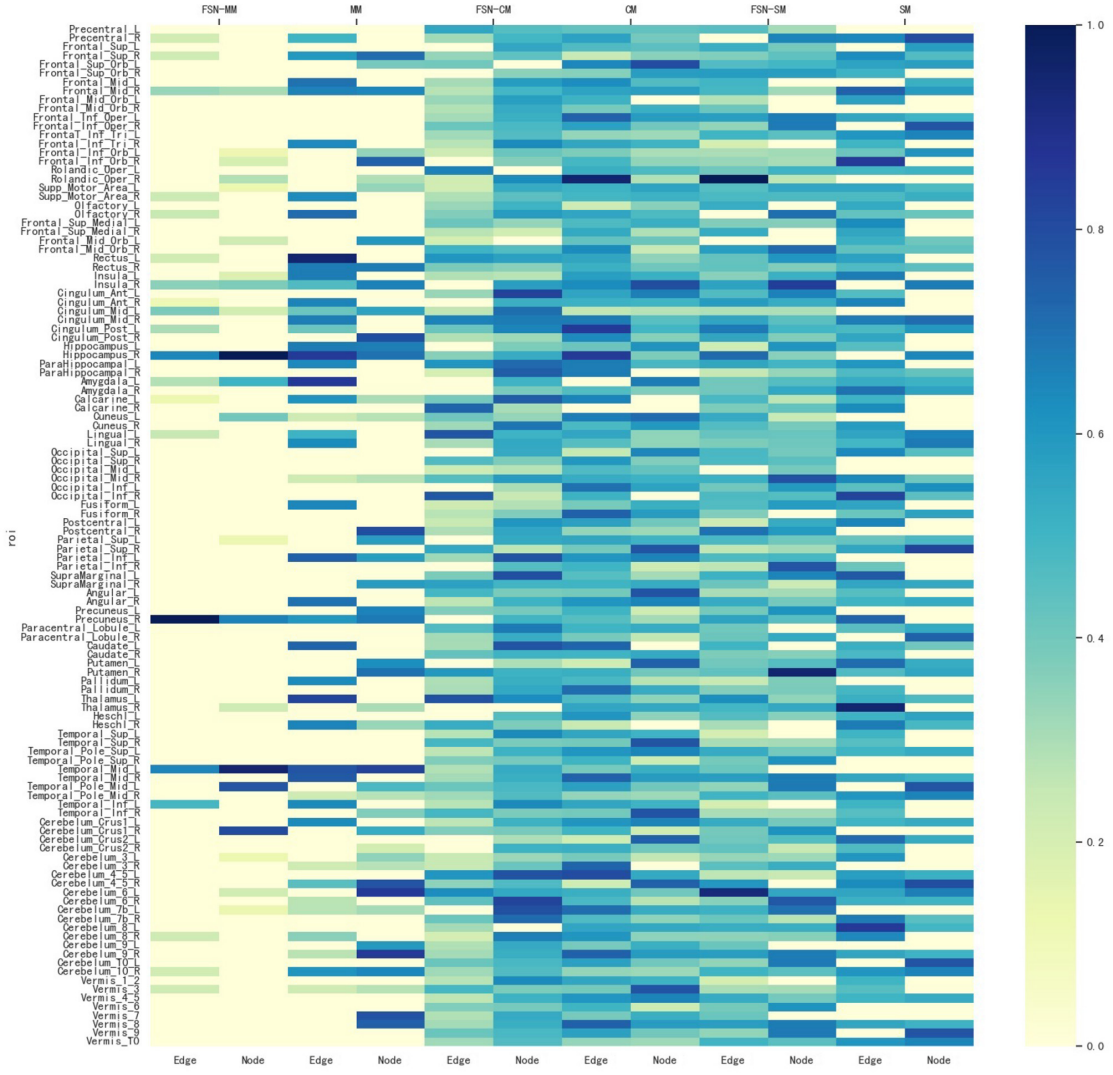
Weight maps for the multi-modalities on 116 ROI associations with THP1 SNP rs1799913 respect to different methods.

To identify ROIs related to MDD, the FSN-MM method identifies consistent ROIs in both node and edge features in our experiments. Figure 4 shows the weight maps for the 116 ROIs associated with the risk SNP TPH1 rs1799913 across multiple modalities. The values of the weights are normalized, where the depth of color in the colorbar represents the magnitude of the ROI weight. In Figure 4, the SM-based and CM-based methods select numerous ROIs, but these ROIs are inconsistent in terms of the node and edge features. Such findings suggest that it might be difficult for researchers to conduct further investigations using these selected ROIs. However, the FSN-MM method can identify sparse and consistent ROIs associated with THP1 rs1799913 by multi-modality imaging data. These identified ROIs, such as the left temporal, right hippocampus and right precuneus regions are consistent with existing research findings and are highly correlated with MDD (Li et al., 2018; Dvorak et al., 2019; Roddy et al., 2019; Wang et al., 2020; Brosch et al., 2022).

In summary, the FSN-MM method tends to select consistent ROIs associated with the risk SNP in multi-modality imaging data, in contrast to methods like SM and CM. This is of significant value for further exploration into the mechanisms of MDD.

### 4.3 Discovery of imaging-genetic patterns in MDD using FSN-MM

#### 4.3.1 Discovery of new potential risk SNPs

The etiology of MDD arises from multiple genetic risk SNPs rather than a single SNP. In the experiment, we examine the relationship between two brain network features and the MDD risk SNP (TPH1 rs1799913). The results show that the FSN-MM method is an effective tool for uncovering novel risk SNPs associated with MDD. In this study, the genotype data contain 5,897 SNPs, and we use the FSN-MM method for each SNP in the entire genotype dataset. Table 5 presents the results. Besides TPH1 rs1799913, 15 other SNPs exhibit a strong relationship with both node and edge features, suggesting that these 15 SNPs might be risk genetic SNPs for MDD.

**Table 5:** Regression performance on multiple SNPs.

**Gene**	**SNP**	**Description**	**References**	**CC (mean** ±**SD)**	

				**Node**	**Edge**
TPH1	rs179995813	Model validation	Jarienė et al., 2018	0.3845 ± 0.0040	0.4037 ± 0.0095
PIK3R1	rs3730089	Supported by literature on MDD and related brain disorders, including schizophrenia and bipolar disorder	Huang et al., 2020	0.3246 ± 0.0169	0.3526 ± 0.0728
DISC1	rs3738401		Zhang et al., 2006	0.3722 ± 0.0024	0.3382 ± 0.0218
COMT	rs4680		Stein et al., 2006	0.3562 ± 0.0398	0.3617 ± 0.0237
HTR2A	rs6311		Smith et al., 2013	0.3255 ± 0.0382	0.3468 ± 0.0218
BDNF	rs6265		Lisiecka et al., 2015	0.3703 ± 0.0383	03608 ± 0.1077
CACNA1C	rs11832738		Liu et al., 2020a	0.3236 ± 0.0867	0.3397 ± 0.0625
CAMK2B	rs11542227	Lacking literature support on MDD and related brain disorders, such as schizophrenia and bipolar disorder	-	0.3204 ± 0.0627	0.3401 ± 0.0583
GALC	rs73312836		–	0.2660 ± 0.0690	0.3166 ± 0.0845
NRG1	rs77493513		–	0.3119 ± 0.0048	0.3033 ± 0.0594
MAPK10	rs1201		–	0.3192 ± 0.0070	0.2601 ± 0.0067
KDSR	rs1138488		–	0.2230 ± 0.0454	0.2158 ± 0.0788
DRD2	rs6279		–	0.2328 ± 0.0768	0.2560 ± 0.0608
LAMA2	rs2229848		–	0.2766 ± 0.0761	0.2867 ± 0.0804
GRIA3	rs550640		–	0.2471 ± 0.1027	0.2729 ± 0.0618
THY1	rs3138094		–	0.2432 ± 0.0288	0.2571 ± 0.0929

Among the 15 SNPs, rs3730089, rs3738401, rs4680, rs6311, rs6265, and rs11832738 are potential candidates that might be associated with MDD. Specifically, the literature (Huang et al., 2020) showed that PIK3R1 rs3730089 was associated with schizophrenia and bipolar affective disorder in the Han Chinese population. Schizophrenia, bipolar affective disorder, and major depressive disorder (MDD) often exhibit shared symptoms such as anhedonia and lack of motivation. Systematic reports (Chen et al., 2017) highlighted the familial aggregation of these three psychiatric disorders, with co-occurrence of any two or even all three disorders in some families. Moreover, evidence from symptomatology and psychopharmacology suggested an intrinsic connection between these three psychiatric disorders. Therefore, PIK3R1 rs3730089 might have served as a risk SNP marker in MDD research. Similarly, Zhang et al. (2006) indicated an association between DISC1 rs3738401 and schizophrenia in the Scottish population; Stein et al. (2006) demonstrated an association between COMT rs4680 and schizophrenia; Smith et al. (2013) suggested that HTR2A rs6311 moderately influenced the severity of depression; Lisiecka et al. (2015) revealed that the allelic variation of BDNF rs6265 led to specific neuro correlates of MDD, which might have been related to different mechanisms of MDD in the two allelic groups and might have had potential implications for patient screening and treatment. Liu et al. (2020a) suggested that the CACNA1C rs11832738 gene variant affected the severity of depression in MDD patients. The six SNPs are substantiated in the literature to be associated with depression, schizophrenia, and bipolar disorder. Consequently, these six SNP markers are pertinent to MDD research and hold referential value (Stein et al., 2006; Zhang et al., 2006; Smith et al., 2013; Lisiecka et al., 2015; Huang et al., 2020; Liu et al., 2020a).

Furthermore, although CAMK2B rs11542227, GALC rs733112836, NRG1 rs77493513, MAPK10 rs1201, KDSR rs1138488, DRD2 rs6279, LAMA2 rs2229848, GRIA3 rs550640, and THY1 rs3138094 exhibit good correlation coefficients through the FSN-MM method, there is no direct medical or biological research supporting the association of these nine SNPs with MDD at this time. We anticipate future studies will validate these findings. We hope that these novel MDD risk SNPs will be confirmed in future research, providing further insights into the etiology of MDD. In total, we have identified 15 new potential SNPs associated with MDD, which may contribute to the study of MDD.

#### 4.3.2 ROI markers identification from sMRI data

Beyond enhancing the performance of correlation analyses, a primary objective of this study is to identify significant imaging phenotypes. For the sMRI data, we analyze six new SNPs with literature support from Table 5. Using five-fold cross-validation performed five times, we obtain average weight values. We select the top five ROIs with the highest weights for each SNP as significant ROI markers. Since the top five ROIs for each SNP are not the same, a combination results in seven unique ROIs. Table 6 displays these seven ROIs and their average weight values.

**Table 6:** The top five ROIs chosen based on the node features from sMRI data for the six SNPs.

**ID**	**ROI**	**Weight**
38	Hippocampus.R	9.12
85	Middle Temporal Gyrus.L	8.97
68	Precuneus.R	7.52
8	Middle Frontal Gyrus.R	2.33
33	Middle Cingulate Gyrus.L	2.30
41	Amygdala.L	2.11
30	Insula.R	1.91

**Figure 5:**
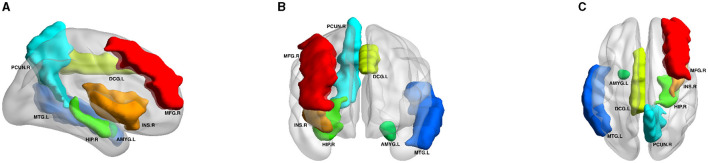
Visualization of the seven ROIs selected by node features in three planes. HIP.R, Hippocampus.R; MTG.L, Middle Temporal Gyrus.L; PCUN.R, Precuneus.R; MFG.R, Middle Frontal Gyrus.R; DCG.L, Middle Cingulate Gyrus.L; AMYG.L, Amygdala.L; INS.R, Insula.R. All volumes of each brain figure are plotted by BrainNet (Xia et al., 2013). **(A)** Axial plane. **(B)** Sagittal plane. **(C)** Coronal plane.

Figure 5 displays the visualization of these seven selected ROIs mapped onto the human brain in all three planes (sagittal, coronal, and axial). In each plane, the color of the labeled brain regions reflects the location of a selected ROI. Notably, most of the selected ROIs are consistent with earlier findings that focus on structural images and identify several diagnostic markers of MDD. Roddy et al. (2019) had shown that MDD patients exhibited bilateral volume reduction in the major hippocampal substructures and had identified the core hippocampal region as a potential marker of MDD progression. Moreover, researchers had found that reduced hippocampal gray matter volume was a common characteristic of MDD patients (Brosch et al., 2022). The bilateral middle frontal gyrus had been shown to exhibit significantly increased amplitude of low-frequency fluctuation (ALFF) in patients with subthreshold depression (Liu et al., 2020a; Zhang B. et al., 2021). As described in the literature (Hagan et al., 2015; Lu et al., 2016), abnormalities in the thalamic structure might have been potential feature markers of early-stage MDD. Compared to healthy controls, MDD patients exhibited reduced gray matter density in the bilateral temporal pole and right superior temporal gyrus (Peng et al., 2011).

#### 4.3.3 ROI markers identification from rs-fMRI data

The functional connectivity between different regions of the brain is represented using a brain network model, where nodes and edges are defined as brain regions and their connections, respectively. In this study, a brain functional connectivity network is constructed using rs-fMRI data. Clustering coefficients are then extracted as edge features and incorporated into each brain region. Consequently, the dimensionality of the obtained edge features is the same as the number of brain regions, with each dimension corresponding to a specific brain region. This approach allows for the identification of relevant ROI markers from rs-fMRI data.

For the edge features in rs-fMRI data, the average weight values are also obtained through five-fold cross-validation with five repetitions, and the ROI with the highest weight among the six SNPs with literature references, is selected as a significant ROI marker, as shown in Table 7, focusing on the prefrontal cortex and hippocampus. Previous research using rs-fMRI data identified key brain region markers for MDD, such as the prefrontal cortex, hippocampus, and temporal gyrus. These markers coincide with the relevant ROIs we've highlighted. MDD patients exhibited abnormal patterns in the prefrontal cortex during rest before starting treatment, suggesting this region could be a potential diagnostic marker for MDD (Li et al., 2018). According to the literature (Sambataro et al., 2014), MDD patients showed enhanced connectivity in the default mode network, especially in the right hippocampus.

**Table 7:** The top one ROI chosen based on the edge features from rs-fMRI data for the six SNPs.

**SNP**	**ID**	**ROI**	**Weight**
rs3730089	68	Precuneus.R	7.40
rs6311	68	Precuneus.R	8.73
rs6265	68	Precuneus.R	8.49
rs11832738	68	Precuneus.R	8.63
rs3738401	38	Hippocampus.R	7.93
rs4680	68	Precuneus.R	8.17

**Figure 6:**
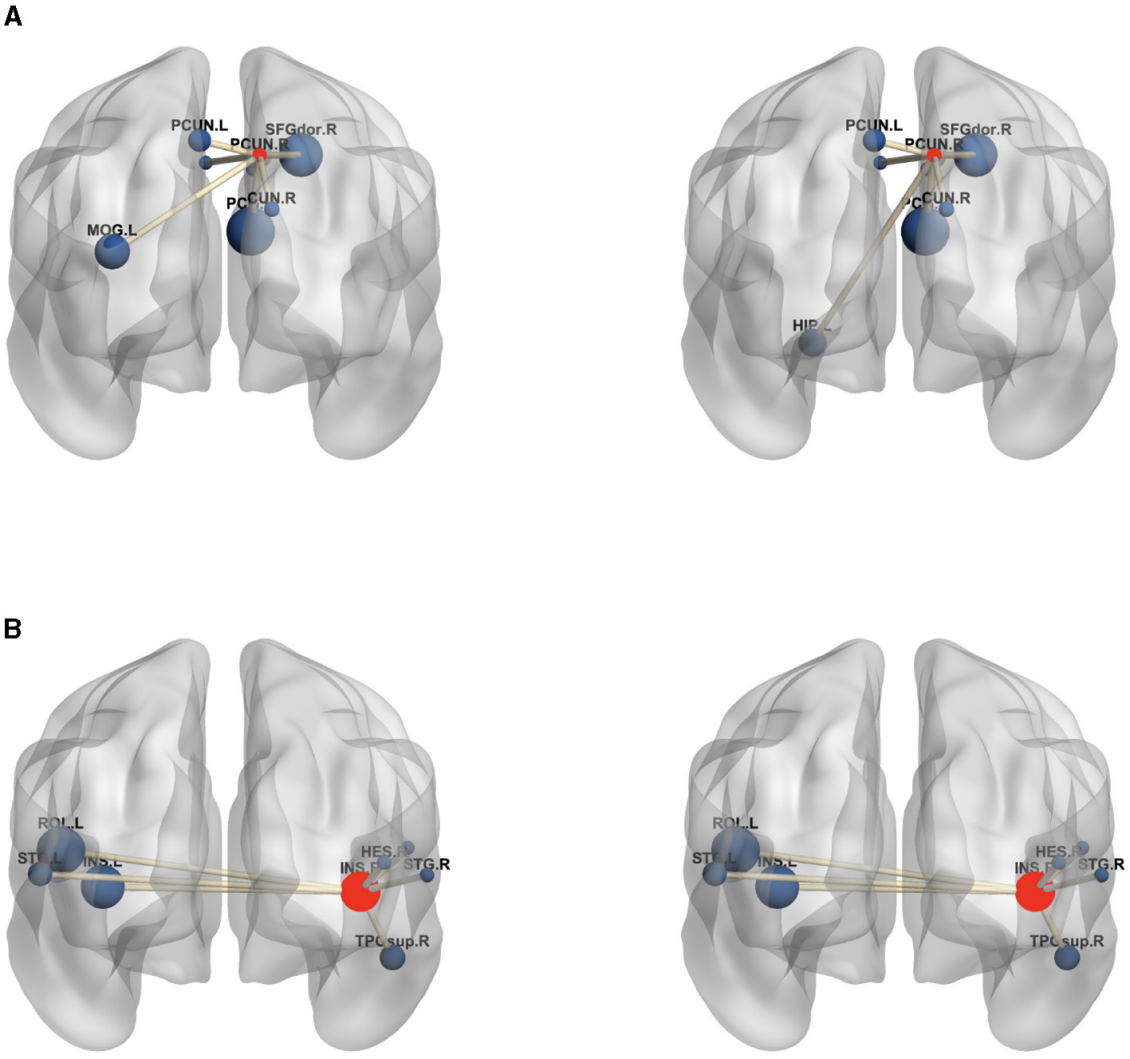
The edges of the maximum weight ROI **(A)** and smaller weight ROI **(B)** on the MDD (left) and HC (right) group. The centroid red node represents the selected ROI, and the blue node denotes the corresponding ROI linked by top seven average connection value edges. **(A)** PCUN.R, Precuneus.R; SFGdor.R, superior frontal gyrus.R; DCG.L, Median Cingulate and Paracingulate Gyri.L; DCG.R, Median Cingulate and Paracingulate Gyri.R; PCG.R, Posterior Cingulate gyrus.R; CUN.R, Cuneus.R; HIP.L, Hippocampus.L; PCUN.L, Precuneus.L; MOG.L, Middle Occipital Gyrus.L; **(B)** INS.R, Insula.R; INS.L, Insula.L; HES.R, Heschl.R; ROL.L, Rolandic Oper.L; ROL.R, Rolandic Oper.R; STG.L, Temporal Sup.L; STG.R, Temporal Sup.R; TPOsup.R, Temporal Pole Sup.R. In the figure, all edges of each brain figure are plotted by BrainNet.

Moreover, to analyze the functional connectivity of the selected brain regions and visualize the differences between MDD and HC on the functional connectivity network, the ROI with the highest weight (right prefrontal cortex) and an ROI with a lower weight (right insula) are selected from the highest weight ROIs corresponding to the six SNPs in Table 7. The average edge values of the functional connectivity networks for the MDD and HC groups are computed separately. Specifically, functional connectivity networks for each participant in the MDD and HC groups are constructed, followed by the computation of the average functional connectivity networks for each group. Lastly, the seven edges with the highest connection values are selected from all the edges within the given ROIs (Wang et al., 2019). Figure 6 graphically displays the first seven edges with the highest average connection values on the highest weight ROI and lower weight ROI. As depicted in Figure 6, the MDD group exhibits significant variations in the highest weight ROI edges compared to the HC group, where the edge connecting the right prefrontal cortex with the left hippocampus is replaced by the edge connecting the right prefrontal cortex with the left cingulate gyrus. The edges of the lower-weight ROI in the MDD group are similar to those in the HC group. These results indicate a strong association between the identified critical brain regions (highest weight ROI) and the pathogenesis of MDD.

#### 4.3.4 Consistent ROI identification from multi-modality imaging data

**Figure 7:**
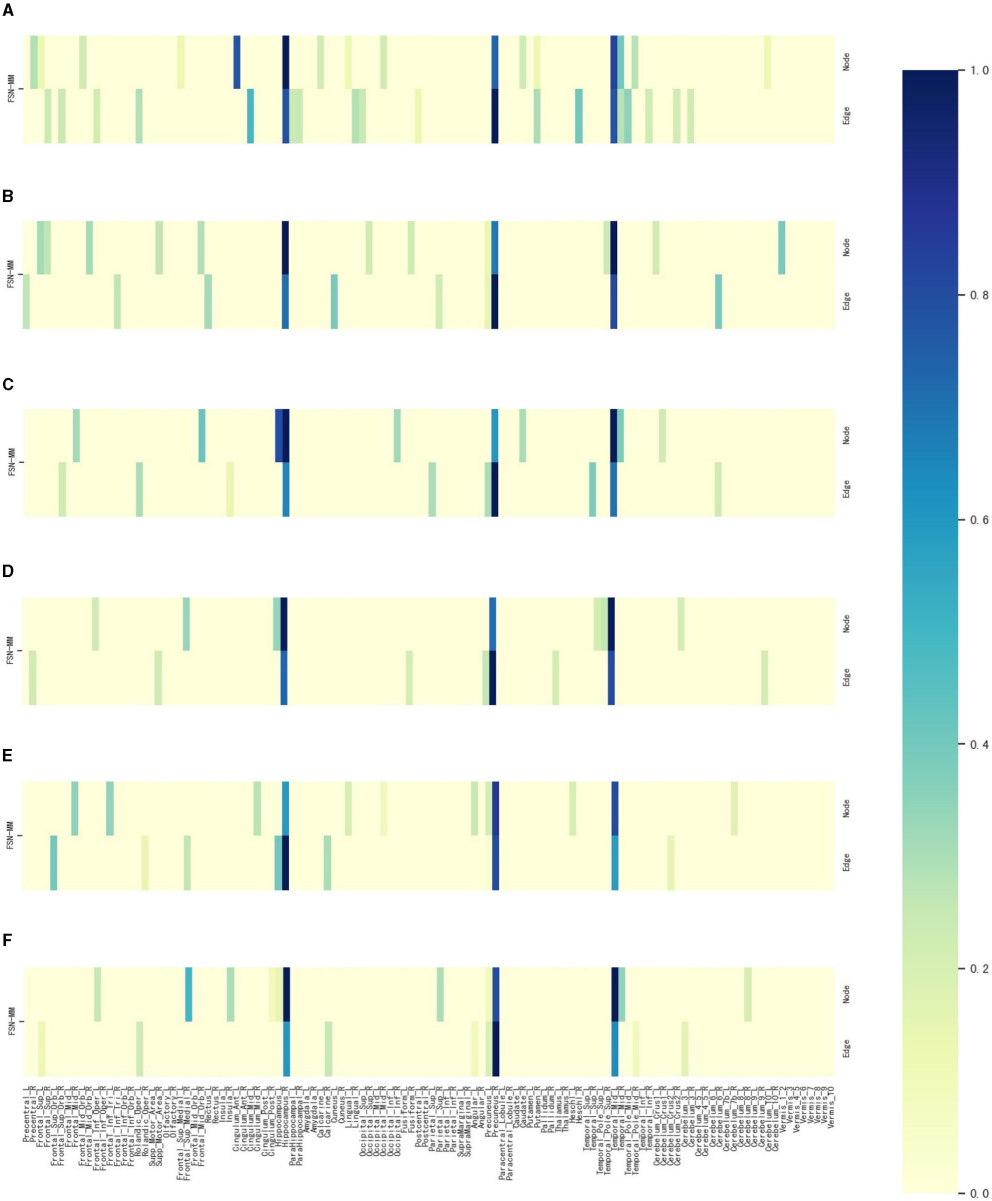
Weight maps for the multi-modalities on 116 ROI associations with six SNPs using FSN-MM. **(A)** rs3730089. **(B)** rs6311. **(C)** rs6265. **(D)** rs11832738. **(E)** rs3738401. **(F)** rs4680.

Figure 7 illustrates the weight maps of the multi-modality data for the 116 ROIs associated with six SNPs. The FSN-MM method successfully identifies sparse and consistent ROIs associated with the six SNPs. ROIs highly correlated with MDD are predominantly located in the left temporal, right hippocampus, and right precuneus regions. These identified ROIs align with previous findings, further validating the accuracy of our research (Li et al., 2018; Dvorak et al., 2019; Roddy et al., 2019; Wang et al., 2020; Brosch et al., 2022).

#### 4.3.5 Consistent ROI identification from multi-modality imaging data for SNPs without literature support

**Figure 8:**
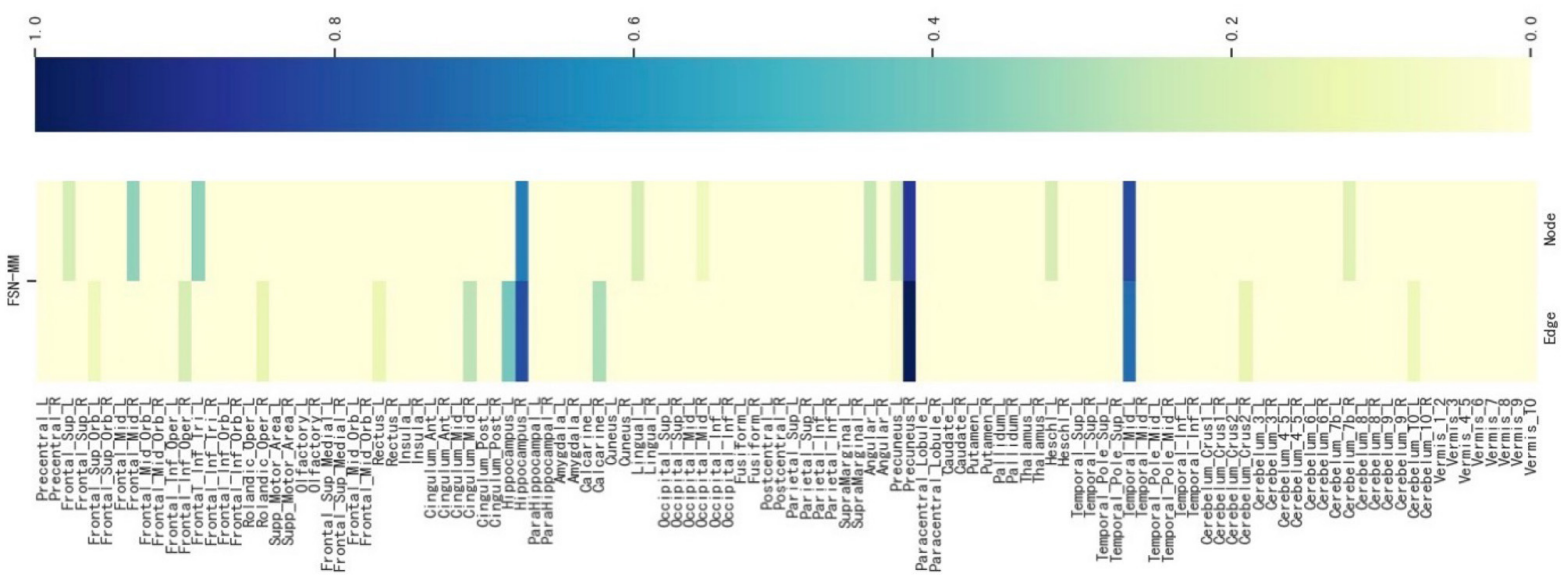
Weight maps for the multi-modalities on 116 ROI associations with LAMA2 SNP rs2229848 using FSN-MM.

We select an SNP (LAMA2 rs2229848) without literature support from Table 5, presenting its weight maps (Figure 8) for the multi-modalities on 116 ROIs. As depicted in Figure 8, the FSN-MM method adeptly identifies sparse consistent ROIs associated with rs2229848. ROIs showing a strong correlation with MDD are still chiefly located in the left temporal, right hippocampus, and right precuneus. This result is consistent with SNPs supported by the literature. While direct medical or biological research supporting the association of these 9 SNPs with MDD is currently lacking, it is anticipated that forthcoming studies will validate these findings, providing valuable insights into the etiology of MDD.

## 5 Discussion

### 5.1 Discriminant and convergence performance of fusion self-expressive network

When comparing the fusion self-reconstructed data with the original data and the self-reconstructed data in Figures 2, 3, we observe a more scattered distribution among different classes and a more concentrated distribution within the same class. This indicates that the fusion self-expressive network generates more discriminative features for the diagnosis information (i.e., HC, MD, or SD).

**Figure 9:**
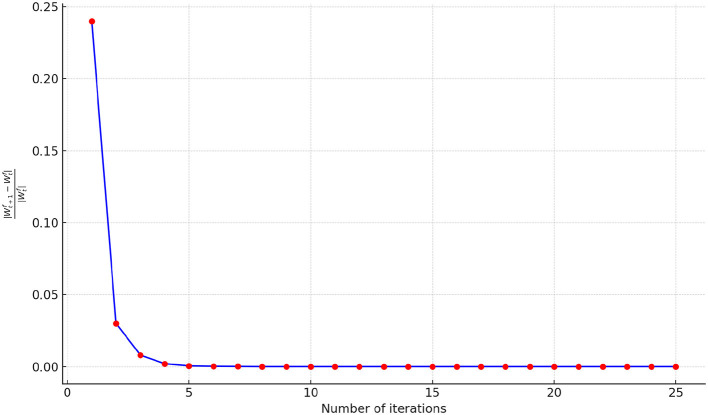
Number of iterations.

To illustrate the convergence performance during the fusion process, we monitor the relative change of parameters in each iteration using the formula $\frac{\left||W_{t+1}^{f}-W_{t}^{f}|\right|}{\left||W_{t}^{f}|\right|}$, where $W_{t}^{f}$ represents the output of the fusion process after *t* steps. The initial iteration *t* is set to 25, and a simple stopping criterion is applied by setting a threshold of 10^−6^. If the relative change falls below the threshold, the optimization procedure terminates. The convergence performance of FSN-MM is illustrated in Figure 9. The relative change value decreases rapidly within the first 10 iterations, signifying the fast convergence of our proposed optimization algorithm. Thus, we set the number of iterations to 10 in our study.

### 5.2 Parameters selection

**Figure 10:**
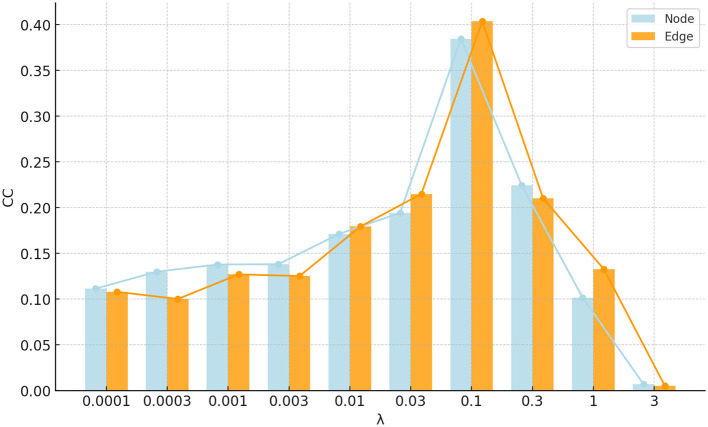
Bar and line graph of correlation coefficients for parameter λ on node and edge features.

The proposed FSN-MM method has only one regularization parameter: the sparsity parameter λ. This parameter is used to balance the relative contributions of the two terms in Equation 8. To investigate the impact of the regularization, the parameter λ was set in the range of 10^−4^, 3 × 10^−4^, 10^−3^, 3 × 10^−3^, …, 1, 3. Figure 10 shows the correlation coefficients of the parameter λ on node and edge features. The performance in the region where 0.03 < λ < 0.3 is pretty good for both node and edge features. This region can be helpful for quickly selecting the optimal value of the parameter λ in future studies.

### 5.3 Atlas selection

The AAL atlas is widely used in many neuroimaging studies due to its comprehensive coverage and clear demarcation of brain regions. To maintain consistency with prior related studies and to make our results comparable, we opted for the AAL atlas. However, choosing different atlases might have a significant impact on the results. Consequently, we also utilized the Brodmann Areas (BA) and the Harvard-Oxford Atlas (HOA) to delineate brain regions and conduct experiments.

**Figure 11:**
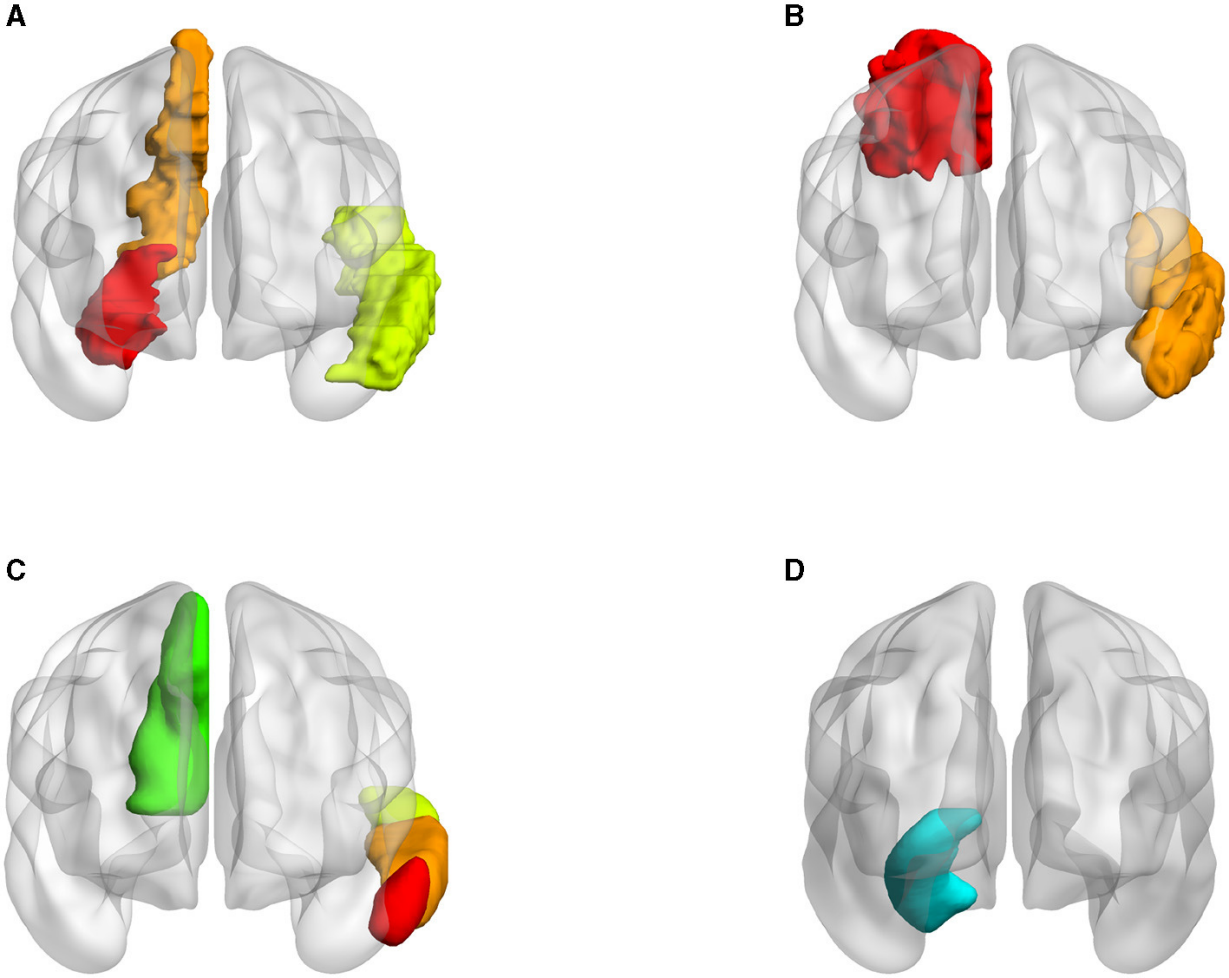
Visualization of ROIs related to MDD in different atlases (all volumes of each brain figure are plotted by BrainNet). **(A)** AAL. **(B)** BA. **(C)** HOA (cortical). **(D)** HOA (subcortical).

Three atlases were tested with SNP rs1799913 respectively. We selected consistent ROIs in both node and edge features. As depicted in Figure 11, in the AAL atlas, the left temporal, right hippocampus, and right precuneus regions are highly correlated with MDD. In the BA, Brodmann areas 7 and 21 are highly correlated with MDD. These two areas largely correspond to the right precuneus and left temporal in the AAL atlas. The hippocampus is not delineated based on Brodmann's divisions since Brodmann's categorizations are primarily based on the cellular structure of the cerebral cortex. However, the hippocampus is a subcortical structure, so it does not directly correspond to a specific Brodmann area. In the cortical and subcortical sections of the Harvard-Oxford Atlas, the regions of the left temporal, right hippocampus, and right precuneus are again shown to be closely correlated with MDD. These three brain regions align closely with the locations identified in the AAL atlas. When experimenting with three atlases, we observed a significant level of consistency in our conclusions.

## 6 Conclusion

In this study, we proposed the FSN-MM method to optimally utilize the internal structure of brain imaging data and diagnosis information for correlation analysis. When we validated using the SNP THP1 rs1799913, our method outperformed other approaches in CC evaluations. Furthermore, we identified consistent and stable ROI biomarkers from the multi-modality phenotype network's voxel node and connectivity edge features. We also identified 15 MDD-associated risk SNPs, of which, six including CACNA1C rs11832738 were supported by the literature. The remaining nine SNPs await future validation.

This study probed the association between a single MDD genetic risk SNP and multi-modality neuroimaging data (sMRI and rs-fMRI). However, this study had limitations. The samples came from only two hospitals, and the sample size was not large enough. Moreover, much of the SNP data was not provided to us for research. We hoped that in the future, more MDD data would be made publicly available for research purposes. Additionally, future research could incorporate other brain imaging modalities like DTI and assess the relationship with multi-locus risk SNPs. Given the advancements in deep learning for biology and medicine, such techniques could tackle brain imaging-genetic associations in depression, potentially enhancing our understanding and treatment of the condition.

## Data availability statement

The datasets presented in this study can be found in online repositories. The names of the repository/repositories and accession number(s) can be found in the article/supplementary material.

## Ethics statement

The studies involving humans were approved by Zhongda Hospital and Xinxiang Hospital. The studies were conducted in accordance with the local legislation and institutional requirements. The participants provided their written informed consent to participate in this study. Written informed consent was obtained from the individual(s), and minor(s)' legal guardian/next of kin, for the publication of any potentially identifiable images or data included in this article.

## Author contributions

MP: Conceptualization, Data curation, Formal analysis, Methodology, Software, Validation, Visualization, Writing—original draft, Writing—review & editing. XL: Writing—review & editing. XH: Writing—review & editing. MW: Writing—review & editing. CX: Writing—review & editing. LZ: Writing—review & editing, Supervision, Writing—original draft. YY: Writing—review & editing.
